# Supplementary material for: IL-13 Modulates Antiviral Effector and Proinflammatory Pathways in Rhinovirus-Infected Pediatric Bronchial Epithelium
Source: bioRxiv. 2025 Dec 8:2025.12.06.692776. Preprint. [Version 1] doi: 10.64898/2025.12.06.692776 (PMC12713584; doi:10.64898/2025.12.06.692776)
Supplement: Supplement 5 [file NIHPP2025.12.06.692776v1-supplement-5.pdf]

bioRxiv preprint doi: https://doi.org/10.64898/2025.12.06.692776; this version posted December 8, 2025. The copyright holder for this preprint (which was not certified by peer review) is the author/funder, who has granted bioRxiv a license to display the preprint in perpetuity. It is made available under aCC-BY 4.0 International license.

SUPPLEMENTAL INFORMATION

Supplemental Methods

RNA Sequencing and Data Processing

The SMART-Seq v4 Ultra Low Input RNA Kit for Sequencing (Takara, San Jose, Calif) was used to create libraries from total RNA. Libraries were reverse transcribed then amplified to generate full-length cDNA amplicons. The NexteraXT DNA sample preparation kit with unique dual indexes (Illumina, San Diego, Calif) created Illumina-compatible barcoded sequencing libraries. A Qubit Fluorometer (Thermo Fisher Scientific, Waltham, Mass) pooled and quantified libraries with subsequent sequencing of pooled libraries on a NextSeq 2000 sequencer (Illumina). Sequencing was done with paired-end 53-base reads and a target depth of 5 million reads per sample. BaseSpace (Illumina) was used to process base calls and quality-trim low-confidence base calls from read ends. Casava (Illumina) was used to deconvolute and convert resulting bcl files to fastq files that were subsequently aligned to the Ensembl human genome (GRCh38, Ensembl 91) via STAR (version 2.4.2a). Gene counts were generated with HTSeq-count (version 0.4.1) using the parameters of “intersection (nonempty)” mode, minimum alignment quality of 20, and otherwise default parameters. PICARD (version 1.134), FASTQC (version 0.11.3), Samtools (version 1.2), and HTSeq-count (version 0.4.1) were used to generate quality metrics. Samples were used if quality metrics showed human aligned counts greater than 1 million mapped reads and a median coefficient of variation coverage less than 0.7. A gene filter was applied to include only protein coding genes that had a trimmed mean of M value normalization count of at least 0.3 in at least 10% of samples. The limma R package (version 4.2.1) function voomWithQualityWeights was used to transform normalized counts to log2 counts per million mapped reads along with observations level weights.

bioRxiv preprint doi: https://doi.org/10.64898/2025.12.06.692776; this version posted December 8, 2025. The copyright holder for this preprint (which was not certified by peer review) is the author/funder, who has granted bioRxiv a license to display the preprint in perpetuity. It is made available under aCC-BY 4.0 International license.

Data Availability

GSE for RNA sequencing data upload to NCBI Gene Expression Omnibus database is pending.

Histopathology

Hematoxylin and eosin (H&E) staining and immunofluorescence were performed on 10% formalin-fixed paraffin-embedded 5-µm sections from transwell membranes oriented en face. Dual immunofluorescence for rabbit alpha tubulin (1:1000 dilution; Invitrogen, #PA5-105102) and mouse MUC5AC (1:300 dilution; ab3649, Abcam) was carried out following citrate pH 6.0 antigen retrieval and serum block; antibodies were incubated for 2 hours at room temperature. Alpha tubulin was developed with donkey anti-rabbit Alexa Fluor 488 and MUC5AC with donkey anti-mouse cyanine (Cy3) (both 1:1000 Jackson ImmunoResearch). Coverslips were mounted using Vectashield fluorescent mounting medium with DAPI (Vector Laboratories). Images were visualized and captured with a digital camera mounted on a Nikon Eclipse 80i microscope using NIS-Elements Advanced Research Software 6.10.01 (Nikon Instruments Inc., Melville, NY).

For scanning electron microscopy (SEM), BECs in transwells were fixed in 2% paraformaldehyde and 2% glutaraldehyde in 0.1M phosphate buffer at 4C°. 0.1M phosphate was used for rinsing and samples were post-fixed in 1% osmium tetroxide overnight at 4C°. Samples were then washed in water, dehydrated with 100% ethanol, and critical point dried. Gold sputter coating was then applied. Samples were imaged using a Thermo Fisher Scientific FEI Apreo VolumeScope scanning electron microscope.

For transmission electron microscopy (TEM), transwells were fixed and dehydrated in the same manner as samples processed for SEM. Briefly, transwells were fixed in 2% paraformaldehyde and 2% glutaraldehyde in 0.1M phosphate buffer and post-fixed in 1% osmium tetroxide, then gradually dehydrated to 100% EtOH. After dehydration, transwell membranes were removed with a biopsy punch tool and transitioned through propylene oxide, gradually infiltrated with

bioRxiv preprint doi: https://doi.org/10.64898/2025.12.06.692776; this version posted December 8, 2025. The copyright holder for this preprint (which was not certified by peer review) is the author/funder, who has granted bioRxiv a license to display the preprint in perpetuity. It is made available under aCC-BY 4.0 International license.

Spurr's low viscosity resin, until finally embedding and curing at 70C° for 24 h. Resin blocks were sectioned with Leica EM UC7 Ultramicrotome at 70nm using a glass knife on formvar-coated 100-mesh copper grids. Samples were then stained with 2% uranyl acetate for 8 min, Reynolds lead citrate for 5 min, then imaged using the FEI Tecnai G2 20 TEM.

bioRxiv preprint doi: https://doi.org/10.64898/2025.12.06.692776; this version posted December 8, 2025. The copyright holder for this preprint (which was not certified by peer review) is the author/funder, who has granted bioRxiv a license to display the preprint in perpetuity. It is made available under aCC-BY 4.0 International license.

Supplemental Figures

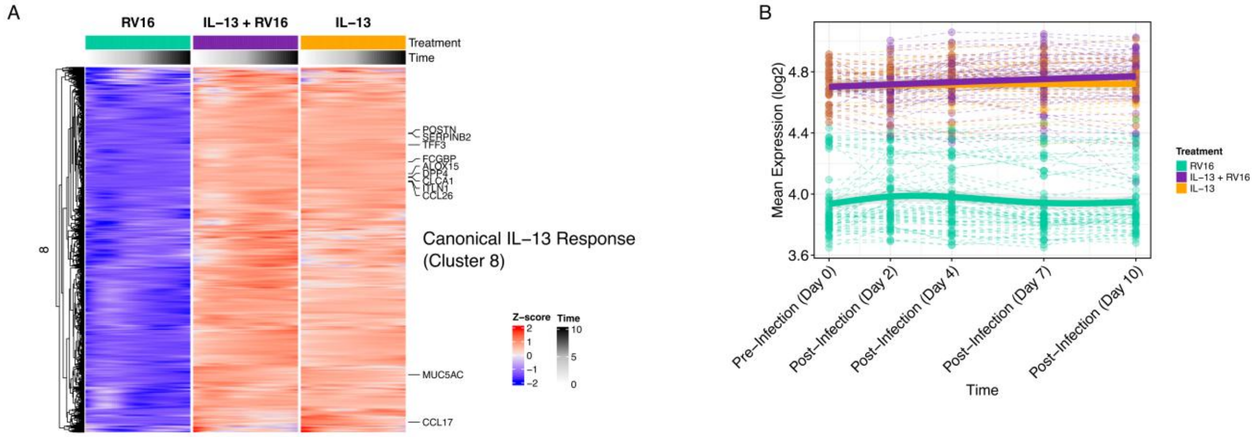

Supplemental Figure 1. Induction of IL-13 canonical response genes A) Heatmap of the cluster annotated as Canonical IL-13 Response identified by hierarchical clustering of GAMM-fitted expression trajectories. Rows represent genes, and columns represent timepoints (0, 2, 4, 7, and 10 days) across the three treatment conditions (RV16, IL-13 + RV16, IL-13). Row values are displayed as Z-scores of mean predicted expression, with red indicating higher relative expression and blue indicating lower expression. Notable IL-13-induced genes are labeled. B) GAMM plot showing the non-linear changes in expression of the Canonical IL-13 Response cluster (cluster 8) across treatments and timepoints.

bioRxiv preprint doi: https://doi.org/10.64898/2025.12.06.692776; this version posted December 8, 2025. The copyright holder for this preprint (which was not certified by peer review) is the author/funder, who has granted bioRxiv a license to display the preprint in perpetuity. It is made available under aCC-BY 4.0 International license.

Supplemental Tables

Supplemental Table 1. Results of the Linear Model showing the differential expressed genes by the treatment groups

Supplemental Table 2. Table of genes in Clusters with Cluster annotations

Supplemental Table 3: Generalized Additive Mixed Models (GAMMs) results comparing expression differences over time between IL-13 treatment groups. Table shows the smoothing terms of time and smoothing interaction term for the effect of IL-13 groups over time.

Supplemental Table 4: Linear Model comparison of viral copy number to the cluster expression.
